# Prx1 cell subpopulations identified in various tissues with diverse quiescence and activation ability following fracture and BMP2 stimulation

**DOI:** 10.3389/fphys.2023.1106474

**Published:** 2023-01-30

**Authors:** Yu Liu, Adrian Ilinski, Louis C. Gerstenfeld, Beth Bragdon

**Affiliations:** Department of Orthopaedic Surgery, Boston University School of Medicine, Boston, MA, United States

**Keywords:** skeletal stem cell, osteogenesis, quiescence, muscle, periosteum, regeneration

## Abstract

The expression of Prx1 has been used as a marker to define the skeletal stem cells (SSCs) populations found within the bone marrow and periosteum that contribute to bone regeneration. However, Prx1 expressing SSCs (Prx1-SSCs) are not restricted to the bone compartments, but are also located within the muscle and able to contribute to ectopic bone formation. Little is known however, about the mechanism(s) regulating Prx1-SSCs that reside in muscle and how they participate in bone regeneration. This study compared both the intrinsic and extrinsic factors of the periosteum and muscle derived Prx1-SSCs and analyzed their regulatory mechanisms of activation, proliferation, and skeletal differentiation. There was considerable transcriptomic heterogeneity in the Prx1-SSCs found in muscle or the periosteum however *in vitro* cells from both tissues showed tri-lineage (adipose, cartilage and bone) differentiation. At homeostasis, periosteal-derived Prx1 cells were proliferative and low levels of BMP2 were able to promote their differentiation, while the muscle-derived Prx1 cells were quiescent and refractory to comparable levels of BMP2 that promoted periosteal cell differentiation. The transplantation of Prx1-SCC from muscle and periosteum into either the same site from which they were isolated, or their reciprocal sites showed that periosteal cell transplanted onto the surface of bone tissues differentiated into bone and cartilage cells but was incapable of similar differentiation when transplanted into muscle. Prx1-SSCs from the muscle showed no ability to differentiate at either site of transplantation. Both fracture and ten times the BMP2 dose was needed to promote muscle-derived cells to rapidly enter the cell cycle as well as undergo skeletal cell differentiation. This study elucidates the diversity of the Prx1-SSC population showing that cells within different tissue sites are intrinsically different. While muscle tissue must have factors that promote Prx1-SSC to remain quiescent, either bone injury or high levels of BMP2 can activate these cells to both proliferate and undergo skeletal cell differentiation. Finally, these studies raise the possibility that muscle SSCs are potential target for skeletal repair and bone diseases.

## Introduction

Bone is a tissue that undergoes continuous remodeling throughout life and possesses the capacity of self-repair after injury or disease-related bone loss. As one of the most common traumatic injuries in humans, fracture imposes a heavy burden on both individuals and society, as it often leads to a decline in quality of life and increased morbidity (Lee et al., 2014). Although the processes of fracture repair are usually biologically robust, approximately 10% of long bone fractures are complicated by delayed healing or non-union (Ekegren et al., 2018) resulting in additional surgical procedures. In addition, heterotopic ossification (HO) is a common complication of trauma, which is characterized by the formation of mineralized bone tissue in soft tissues resulting in stiffening of joints, pain, and impaired movement (Bragdon et al., 2017; Meyers et al., 2019). Therefore, there is a need to develop therapeutic strategies to accelerate normal physiological bone repair as well as to understand the mechanisms behind dysfunctional bone formation like HO. As the appropriate recruitment of skeletal stem cells/progenitor cells is needed for successful bone repair, targeting stem cells/progenitors offers a therapeutic strategy against skeletal dysfunction (Caplan and Bruder, 2001).

To date there is a lack of consensus of cell markers that are unique to SSCs, partially due to the heterogeneity of SSC populations. Advances with genetic mouse models have revealed several markers that can be used to define various populations of SSCs, including Nestin, LepR, Mx1, αSMA, Periostin, Cathespin K, Cxcl12, Prx1 (Méndez-Ferrer et al., 2010; Park et al., 2012; Greenbaum et al., 2013; Zhou et al., 2014; Debnath et al., 2018; Duchamp de Lageneste et al., 2018). Published work along with our studies showed Prx1 expressing cells connote a multi-potential postnatal SSC population that retained its embryonic tissue specification, localized to multiple sites such as bone marrow, periosteum, and muscle within the appendicular regions and contributes to homeostatic maintenance, fracture repair, and HO (Batista et al., 2019; Julien et al., 2021; Bragdon et al., 2022).

Although stem cells from multiple tissue sites can contribute and support fracture repair, growing evidence indicates there is functionally different abilities to support bone regeneration. Many studies leading to the characterization of SSCs rely on bone marrow stromal cells (BMSCs), however, BMSCs may have limited contribution to endogenous skeletal repair (Colnot, 2009). More recently, several studies have suggested the periosteum or the periosteal stem cells (PSCs), rather than bone marrow, as a major cellular contributor to bone regeneration after fracture (Colnot et al., 2012; Ortinau et al., 2019), and exhibit high bone regenerative potential compared to BMSCs in mice models (Debnath et al., 2018). Even with the periosteum, data suggest that osteoblastic differentiation potential differs by location: calvaria periosteum showed less osteogenic potential than tibia (Bilkay et al., 2008). These observations provide evidence that tissue site differences also contribute to the diversity of SSCs. Accumulating evidence is also demonstrating that muscle may contribute to bone regeneration by providing stem cells/progenitors (Schindeler et al., 2009; Lemos et al., 2015). Other than satellite cells, recent studies report the existence of skeletal muscle resident mesenchymal progenitor cells exhibiting multilineage potential and have been shown to express a similar marker profile with BMSCs (Gharaibeh et al., 2008; Wosczyna et al., 2012).

Taken together, these studies indicate the periosteum, muscle and bone marrow contain pools of skeletogenic stem cells/progenitors with distinct functional potentials that vary with the tissue environment. In this study, the Prx1 skeletogenic stem cell populations were isolated from the periosteum and muscle of transgenic mice and transplanted to compare functionality and identify possible regulatory mechanism(s) for bone formation. The heterogeneity between tissue sites and within each tissue was determined with at least three subpopulations identified within each tissue site. These results showed varied proliferation of the Prx1-SSCs derived from the between tissue sites. Using BMP2 and fracture as models of bone formation, the results suggest that the Prx1-SSCs of the muscle are quiescent but when active can contribute to bone formation. This cell population maybe used for future regenerative therapies.

## Methods

### Animals

The wild type C57BL/6J (B6, Jackson Laboratory, Stock 000,664) and transgenic mice, Prx1-CreER/Ai14/Rag1, were used for this study. The Prx1-CreER/Ai14/Rag1 mice was previously described (Bragdon et al., 2022), briefly the strain was established by crossing the Ai14 (007,914, B6. Cg-Gt (ROSA)26Sor^tm14(CAG-tdTomato)Hze^/J), with Prx1-Cre/ERT2-EGFP (provided by Dr. Shunichi Murakami (Kawanami et al., 2009)) and with the Rag1 (002,216, B6.129S7-Rag1^tm1/MOM^/J). These transgenic Ai14 reporter animals express CreER under control of the Prx1 promoter, labeling Prx1 cells and their progeny with dTomato. Experiments included both male and female mice aged 8–12 weeks. All animal studies were approved by the Institutional Animal Care and Use Committee at Boston University (IACUC) and housed and bred at the BU Animal facilities under standard conditions.

### Tamoxifen

Tamoxifen (T5648, Sigma, 100 mg/mL) was prepared in corn oil (Sigma), sterile filtered (25 mM syringe filter), and stored at −80°C. Mice were injected with two doses of tamoxifen (10 mL/kg) intraperitoneally with a gap of 48 h between injections. Days post injection was calculated from the last tamoxifen injection.

### Isolation of primary cells

Three days following tamoxifen treatment, bone marrow, periosteum, and muscle-derived cells were harvested from hindlimbs of Prx1-CreER/Ai14/Rag1 mice. Soft tissue was carefully removed from the femur and tibia not to disrupt the periosteum. The skeletal muscles surrounding the femurs were dissected from tendon to tendon. In a Petri dish with ice-cold PBS, fat and tendon were removed, and tissues were minced using razor blades. Then the bone epiphyses were removed, and bones were flushed with basal medium (aMEM, 10% FBS, 1% Anti-anti) to isolate bone marrow-derived cells (Bragdon et al., 2015). Finally, periosteum was removed from the bone surface using a dissecting microscope and by scrapping the bones. Both the periosteum and muscle required digestion to get individualized cells. The digestion buffer consisted of αMEM (12,571–063, Gibco), Collagenase P (1 mg/mL, 11213873001, Roche), and DNase I (2 units/mL, 04716728001, Roche). Muscle was allowed to be digested for 1 hour at 37°C with agitation, while the periosteum was digested for 20 min using the same conditions. Digested tissues were then pipetted and filtered through 70-µm (22363548, Fisher Scientific) and 40-µm (22363547, Fisher Scientific) nylon mesh strainers. Cells were centrifuged for 5 minutes at 230 g and re-suspended in basal medium.

### Fluorescence assisted cell sorting

Cell sorting was performed on MoFlo Astrios (Beckman Coulter Life sciences) located in the Flow Cytometry Core at Boston University. Beads (01–2222-42, Thermo Fischer) were used for initial compensation set up and FMO controls were used to determine background level of each color. Sorted cells were analyzed in the forward scatter channel (FSC) to assess cell size. Data are presented as representative histograms. Antibodies for FACS included CD45-PECy7 (1:400, 25–0451-82, Invitrogen), CD105-APC (1:100, 120,414, BioLegend), CD200-BV421(1:50, 565,547, BD Biosciences). Directly following primary cell isolation, cells were washed with the sorting buffer (PBS, 2% FBS and 1 mM EDTA). Cells were then incubated on ice in the dark for 20–30 min with antibody solution (combination of antibodies prepared in sorting buffer). After washing cells 2–3 times with sorting buffer, cell viability marker, DAPI (0.2ug/mL, D1306, Invitrogen) was added before sorting. Three distinct subpopulations were isolated by this sorting strategy. They were P1 (Prx1+ CD45^−^ CD105- CD200+), P2 (Prx1+ CD45^−^ CD105- CD200-) and P3 (Prx1+ CD45^−^ CD105+ CD200 ^variable^).

### Cell culture and in vitro differentiation

Sorted cells were plated at a concentration of 2000–4000 cells/well in 24 well plate (3527, Corning) in complete Mesencult Medium (05,501, Stem Cell Technologies) with stimulatory supplements (05,502, 05,500, Stem Cell Technologies). Half of the medium was changed on day four after initial plating followed by full medium changes every two to 3 days. Digital photographs of stained cell within tissue culture wells were taken from a fixed distance on a light box for illumination.

Osteogenic differentiation. Once cells reached confluency, cells were cultured in osteogenic differentiation medium (05,504, Stem Cell Technologies). Medium was changed every two to 3 days for 3 weeks. Cells were washed with cold PBS three times and fixed with 2% PFA for 20 min at room temperature. Cells were then washed with distilled water and stained with alizarin red solution (TMS-008-C, Sigma) at room temperature for 30 min. Cells were washed thoroughly with distilled water for at least five times before imaging.

Adipogenic differentiation. Confluent cells were cultured in adipogenic differentiation medium (05,507, Stem Cell Technologies) for 2 weeks with medium changes every two to 3 days. Cells were fixed with 2% PFA for 20 min at room temperature. After fixation, cells were washed with PBS and stained with oil red O solution (3:2 solution with water, O1391, Sigma) at room temperate for 30 min to visualize lipid droplets.

Chondrogenic differentiation. Cells were seeded at 110^5^ cells in 5 μl droplet of complete Mesencult Medium to create a micromass. Two hours after initial plating, chondrogenic differentiation medium (A10071-01, Stem Pro) was added to culture cells. The medium was changed every two to 3 days for 2 weeks and cells were fixed with 2% PFA and rinsed with PBS. Cells were then stained with 1% alcian blue solution (TMS-010-C, Sigma) for 30min, washed three times with 0.1N HCL and then PBS.

### Surgical models

All mice were anesthetized using 4% Isoflurane for induction and maintained at 2%. Hair was shaved, and skin was prepped with betadine. For all surgeries, fascia and skin were sutured using 6–0 and 5–0 plain gut resorbable sutures, respectively. All animals were given Buprenex (0.01 mg/kg) for immediate postoperative pain management, and Baytril (0.01 mL) for infection prophylaxis by subcutaneous injections at time of surgery which continued for 48 h.

#### Implantation of gelatin sponge

The absorbable gelatin sponge (Gelfoam 09–0353-01, Pfizer) was implanted using the same surgical technique previously described (Bragdon et al., 2017). In brief, the sponge was cut to be 1 cm squared and wetted with sterile PBS with various amounts (0-5ug) of bone morphogenetic protein 2 (BMP2, 355-BM, R&D systems). Prx1/Ai14/Rag1 mice received the implants at post-tamoxifen injection day three. The femur was exposed, and the muscle was bluntly separated from the femur. The gelatin sponge was implanted along either the periosteum surface near the mid-diaphysis or within a muscle pouch between the medial gluteal and femoral quadriceps muscle. The fascia was sutured with 6–0 plain gut resorbable sutures (003–2490, ACE Surgical Supply Co) followed by closing of the skin with 5–0 plain gut resorbable sutures.

#### Transplantation

Three days after sponge implantation, the donor mice were euthanized by carbon dioxide inhalation followed by cervical dislocation and the sponge was aseptically removed from the donor. The sponge was then transplanted to the periosteum surface or intramuscular pouch of the C57BL/6J host mouse using the same approach as described above.

#### Closed stabilized fracture

Closed stabilized transverse fractures were generated at the mid-shaft of the right femur (Lybrand et al., 2015). Briefly, the animals were positioned in a supine position and a small incision was created medially at the knee. The patella was moved to the lateral side and a needle syringe was used to ream the bone marrow. A stylet of a 25-gauge spinal needle was then placed into the medullary canal and cut to size. The patella was placed back into position and the incision was closed. A closed transverse fracture was created using the Bonnares and Einhorn method (Bonnarens and Einhorn, 1984; de Giacomo et al., 2014) and the fracture device (Marturano et al., 2008). Following fracture, the sponge was transplanted adjacent to the fracture site.

### Plain film radiography

At time of euthanization, plain-film radiographs were taken using Faxitron MX-20 Specimen Radiography System (Tucson) at 30 kV for 40 s. DF-50 Ultra-speed dental X-ray film (166–6163, Carestream Health) was used. Film was developed using a Konica Minolta SRX-101A film processor (Wayne).

### MicroCT

MicroCT imaging and analysis was performed using post-operative day 24 fractures. Samples were scanned with the Scanco Medical μCT 40 Scanner. The sample was placed in a 20.5 mm conical tube and scanned at 70 kVp and 114 μA with an integration time of 200 m. Reference lines were adjusted manually on individual bones to include the entire callus area. Scans were manually contoured to analyze the fracture and the implant in the muscle using SCANCO Medical software. The threshold used was consistent with previous analysis and was set to detect the presence of mineralized bone (Wigner et al., 2010). Transverse images were reconstructed digitally to generate a 3D image of the callus.

### Histology

Freshly harvested femurs and tibias were fixed with 4% paraformaldehyde (P6148, Sigma) at 4°C for three to 4 days. Samples were washed with PBS and decalcified with 14% Ethylenediaminetetraacetic Acid (EDTA, 6381–92-6, Acros Organics) for 1 week at 4°C. Samples were incubated with 7.5% sucrose/PBS solution followed by 30% sucrose/PBS, then incubated in a 1:1 mixture of 30% sucrose/PBS and Tissue Plus Optimum Cutting Temperature (OCT, 4585, Fisher Healthcare) compound. Each step of sucrose wash was performed on a rotor at 4°C until the samples sank to the bottom of the tube. Samples were embedded using OCT and frozen blocks were preserved at −80°C. Tissues were cut using a Reichert Jung Cryocut 1800 (Wetzlar, Germany) with sections being 8–10 µm thick. Safranin O/Fast green staining was performed to visualize bone and cartilaginous tissue.

### Immunofluorescence

Immunofluorescent staining was done on sections adjacent to Safranin O. Frozen sections were thawed at room temperature and washed with 1X PBS to remove OCT. Sections were blocked for 1 hour with 5% FBS in PBS (blocking buffer). Dilutions of primary antibodies were freshly prepared in antibody dilution buffer (1% BSA in 1X PBS). Sections were incubated with primary antibody at 4 °C overnight. Secondary antibodies Alexa Fluor 488 anti rabbit (1:1000, A21206, Molecular Probes) was added to the sections for one to 2 hours at room temperature protected from the light. Sections were washed with PBS and mounted using Prolong Gold Antifade Reagent with Dapi (P36935, Invitrogen). Controls were performed following the same procedure above, but the primary antibody was absent (Figure S3). Antibodies used: Osterix (1:100, ab22552, Abcam), ColX (1:1000, ab58632, Abcam), Ki67 (1:100, ab16667, Abcam). For the labeling of Osterix, Triton X-100 (0.3%, T8787, Sigma) was added to the blocking buffer and antibody dilution buffer. Images were collected within 24 h using an Olympus BX51 (OlympusAmerica, Inc.) with the 10x and/or ×20 objective and the cellSensDimension software (version 4.1.0.0, Olympus America). At least 10 sections per sample were analyzed. For cell counts, at least 3 sections were selected from each group, and 10–12 images were taken at ×20 magnification and counted as region of interest (ROI). The number of cells per ROI were counted by CellProfiler.

### Bulk RNA-sequencing

Bulk RNA-sequencing (RNA-seq) was performed on sorted P1, P2, P3 populations isolated from bone marrow, periosteum and muscle of mouse femur and tibia. Sample QC, library preparations, sequencing reactions, and initial bioinformatics analysis were conducted at GENEWIZ, LLC (South Plainfield, NJ, United States of America). Samples were sequenced on the Illumina HiSeq instrument using HiSeq Control Software. Raw sequence data (.bcl files) generated from Illumina HiSeq were converted into fastq files and de-multiplexed using the Illumina bcl2fastq v. 2.17 program. One mismatch was allowed for index sequence identification.

Fastq file quality was evaluated with FastQC, followed by read trimming using Trim Galore. STAR was used to generate an alignment (.bam) file. DESeq2 was used for counts quality control and to generate differential gene expression data. Genes with adjusted *p* values less than 0.05 and absolute log_2_ fold changes greater than 1.5 were called as differentially expressed genes (DEGs) for each comparison. A PCA analysis was performed using the “plotPCA” function within the DESeq2 R package. The heat map was generated using heatmap.2 in the R gplots package or Morpheus online tool, where the expression values were normalized per gene over all samples. The GOcircle function from the GOplot R package was used to generate circular plots of representative GOs.

### ATP measurement

ATP levels of freshly isolated and sorted primary cells were measured using an ATP colorimetric assay kit (MAK190, Sigma) according to the manufacturer’s instructions. Briefly, cells were sorted (P1, P2, and P3) and re-counted with a hemocytometer. Approximately 100,000 cells were used to quantify ATP levels from each tissue and subpopulation. Same number of cells was used for the analysis, and quantitation was done from four independent replicates.

### Statistics

Data are presented as mean ± standard deviation (s.d.) and were obtained from at least three samples for each group. Statistical significance was reported from GraphPad Prism v9.3.1. In all experiments, *p* values < 0.05 were considered significant.

## Results

### Gelatin sponge implantation model to recruit cells and induce ectopic bone

**FIGURE 1:**
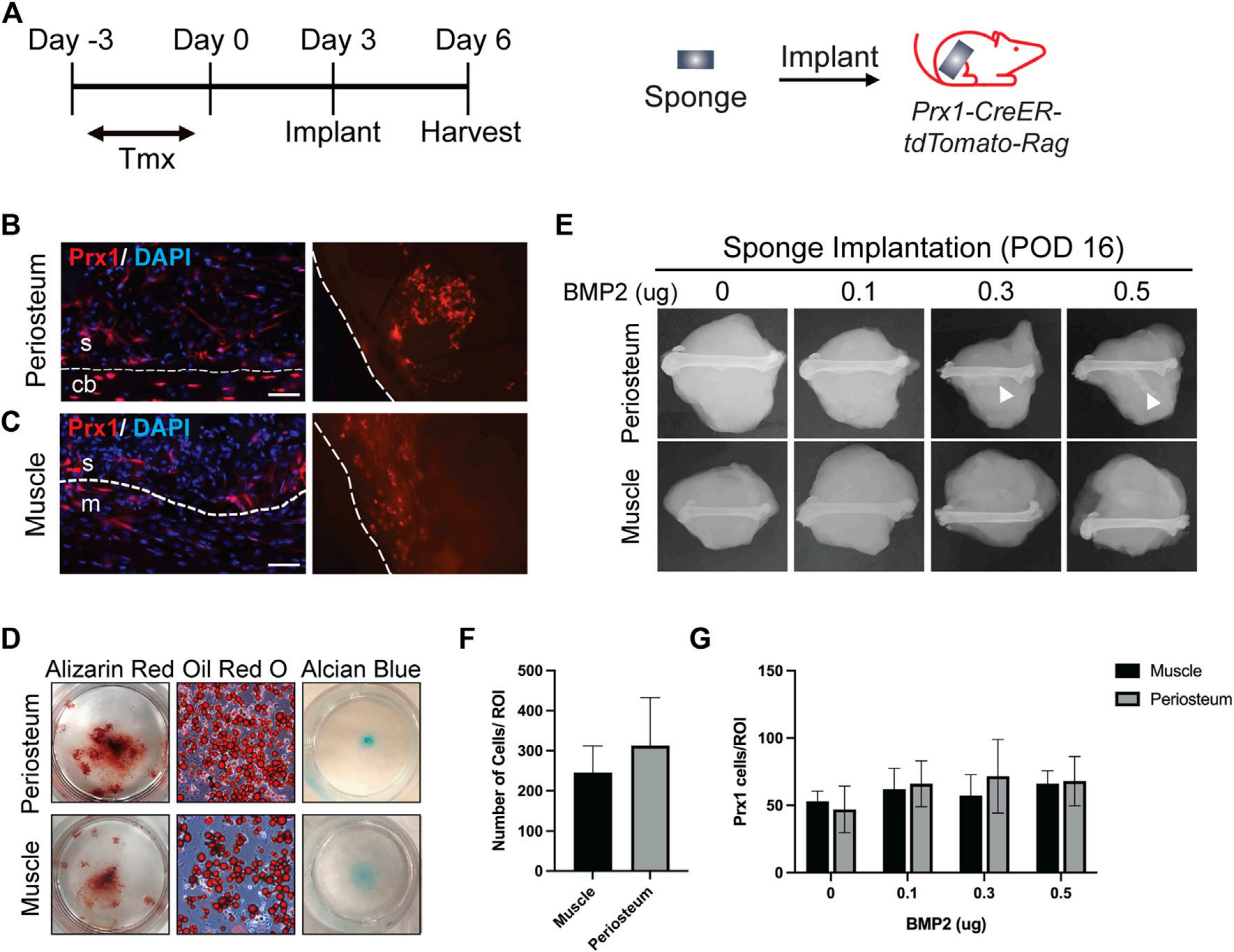
Site Specific Stem Cell Retrieval for *In vitro* and *In Vivo* Assessment of Differentiation Potential **(A)**. Schematic representation of sponge implantation in hindlimbs of Prx1-CreER-tdTomato-Rag donor mice. **(B–C)**. Prx1-tdTomato expression in gelatin sponges that were implanted on the periosteum surface **(B)** or intramusclar pouch **(C)** of Prx1CreER-GFP/Ai14/Rag1 mice. Sponges were harvested after 3 days of implantation. The left images are representative IF staining of the sponges along with surrounding tissues. Right images show tdTomato expression in the sponge that were directly examined under the fluorescent microscope. The sponges are outlined by the dashed lines. Red: tdTomato, blue: DAPI. S = sponge; cb = cortical bone; m = muscle. Scale bars = 50 µm. **(D)** Representative images of *in vitro* differentiation of cells isolated from the sponges implanted on the periosteum or muscle. Cells were cultured in osteogenic, adipogenic, and chondrogenic inducing media to determine differentiation potential. Shown by alizarin red S (osteogenic), Oil red O (Adipogenic), and alcian blue (chondrogenic) staining. **(E)**. Plain film radiography of sponges implanted on the periosteal surface or in the intramuscular pouch of the femur, supplemented with various amount (0–0.5 µg) of BMP2. Sponges were harvested at post-implantation day 16, only the periosteum implants with higher concentration of BMP2 (0.3μg and 0.5 μg) induced ectopic bone (white arrowhead). **(F)**. Quantification of total cells in the sponges from muscle and periosteum. **(G)**. Quantification of Prx1-derived cells tagged with tdTomato in the sponges from muscle and periosteum supplemented with various amount (0–0.5 µg) of BMP2. Data are mean ± s.d.; 3-4 independent experiments.

In order to retrieve local cells and directly compare the regenerative potential of cells from different tissue origins *in vivo*, we adapted a previously published method that had been used as a carrier for BMP2 delivery (Bragdon et al., 2017). A gelatin sponge was implanted on either the periosteum or within an intramuscular pouch and used as a method to recruit local cell populations into the sponge. We first assessed cell recruitment and multipotency of the retrieved cells. Prx1/Ai14/Rag mice were used to fluorescently tag the Prx1 expressing cells and at 3 days post-implantation, the sponges were harvested, and histological analysis was performed (Figure 1A). Results showed Prx1-derived cells tagged with tdTomato as well as other cell populations were recruited to the sponges *in vivo* (Figures 1B,C). Cell retrieved within the gelatin sponges could then be successfully removed and cultured *in vitro* for 3–4 weeks with differentiation media to investigate cell differentiation potential. Results showed cells isolated from the gelatin sponges from both locations were able to differentiate toward the osteoblast, chondrocyte, and adipocyte lineages (Figure 1D). This analysis suggests that the gelatin sponge can be used as an efficient model to recruit local cells and maintain the multipotency of cells.

Since ectopic bone can be induced with BMP2, various amounts of BMP2 were added to the gelatin sponge at time of implantation to assess recruitment of stem cells to the site of implantation and the capacity to form ectopic bone at the differing tissues sites. Ectopic bone was not detected in the periosteal implants with 0 µg or 0.1 µg of BMP2, however it was detected with 0.3 µg and 0.5 µg of BMP2. In contrast, no ectopic bone was found within the muscular implanted gelatin sponges with 0–0.5 µg BMP2 (Figure 1E). In both tissues, similar total number of cells were recruited to the sponges at the time of harvest (Figure 1F). To confirm that BMP2 does not alter Prx1 cell recruitment, Prx1 cells were counted within the gelatin sponge. Gelatin sponge implants at both the periosteal and muscle sites showed similar numbers of recruited Prx1-derived cells (Figure 1G). This indicates that the ability of BMP2 to induce ectopic bone is not due to cell recruitment of these cells into the sponge but primarily due to differentiation potential that BMP2 has, inherent to the site of implantation. Based on these results, the gelatin sponge implant along with BMP2 was used as a screen for examining the abilities of Prx1-derived cells to form bone.

### Differences in the sensitivity of skeletal muscle-derived and periosteum-derived Prx1 to BMP2

Stem cell proliferation and differentiation is highly regulated by external signals, the stem cell niche, and intrinsic factors. It is not clear if the Prx1 cell populations of the muscle and periosteum have similar *in vivo* responses to external stimuli or are intrinsically regulated. Transplant studies using the ectopic bone model were conducted to elucidate functional differences between the muscle and periosteal-derived Prx1 cells. Briefly, 0.5 µg of BMP2 were added to the gelatin sponges and implanted either on the periosteal surface or within the muscular pouch of the Prx1/Ai14/Rag mice. Following 3 days of cell recruitment, the sponges were then transplanted to the wild type B6 host mice, allowing for tracking of the tdTomato tagged Prx1-derived cells from the donor mice into the tissues that formed within the recipient mice.

**FIGURE 2:**
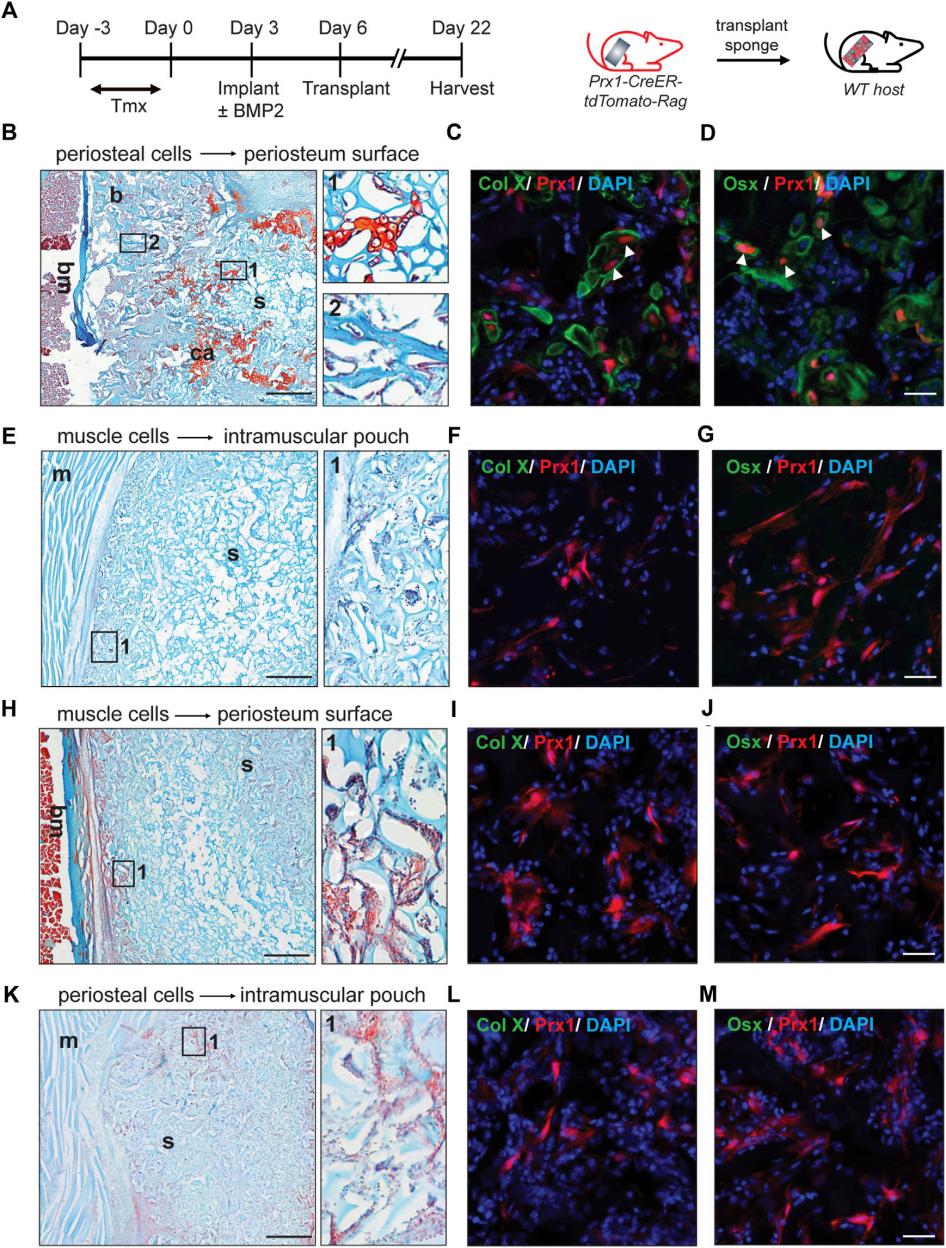
Reaction of periosteal and muscle-derived cells to 0.5 µg BMP2 upon transplantation to the same or different environment. **(A).** Timeline and schematic representation of sponge implantation in hindlimbs of Prx1-CreER-tdTomato-Rag donor mice and subsequent transplantation to B6 host mice. (*n* = 3–5) **(B–D)**. Results of periosteual cells from Prx1 reporter mice transplanted to the periosteal surface of wild-type hosts. **(B)** longitudinal section of the sponge and femur, stained with Safranin O. Right: Enlarged view of boxed regions (1 and 2) shows cartilage and bone formation. **(C–D)**. High magnification of adjacent section in boxed areas were immunostained with cartilage and osteoblast marker. White arrowheads indicate co-labeling of Prx1 derived cells (red) with Collagen X (green) and Osterix (green), nuclei were labeled with DAPI (blue). **(E–G)**. Results of muscle cells from Prx1 reporter mice transplanted to the intramuscular pouch of wild-type hosts. Right: Enlarged view of boxed region 1, shows no bone or cartilage formation. **(F–G)**. Adjacent section immunostained with Collagen X and Osterix (green). **(H–J)**. Results of muscle cells from Prx1 reporter mice transplanted to the periosteum surface of wild-type hosts. **(H)** longitudinal section of the sponge and femur, stained with Safranin O. Right: Enlarged view of boxed regions. **(I–J).** Adjacent section immunostained with Collagen X and osterix (green). **(K–M)**. Results of periosteum cells from Prx1 reporter mice transplanted to the intramuscular pouch of wild-type hosts. Right: Enlarged view of boxed region 1. **(L–M)**. Adjacent section immunostained with Collagen X and Osterix (green). Scale bar = 500 μm for Safranin O, 25um for IF staining. Bm = bone marrow; b = bone; ca = cartilage; s = sponge; m = muscle. Images are representative of 3-5 independent experiments.

First, gelatin sponges were transplanted from the donor to the corresponding same tissue site of the host (periosteum cells transplanted to the periosteum or muscular cells transplanted to the muscle, Figure 2A). At day16 post-transplantation, cartilage and bone formation were found in periosteum sponge, as showed by Safranin O staining and x-ray. (Figure 2B, S1). Specifically, the tdTomato tagged Prx1-derived cells survived transplantation and contributed to the bone formation. Immunofluorescence confirmed that these cells differentiated into osteoblasts and chondrocytes (Figures 2C,D). Various amounts of BMP2 were also used, with 0.1 µg of BMP2 being the lowest which was able to induce chondrogenic differentiation of periosteal-derived Prx1 cells at the time of harvest (Figure S2a). However, bone formation was not detected in the muscle transplants, which was similar to the previous results of gelatin sponge implantation with BMP2 (Figures 2E–G, S1, S2b).

Next, the gelatin sponges were transplanted to a different tissue site of wild-type hosts. The muscle implant of the donor mice was transplanted to the periosteum surface of the wild type B6 host mice. Although haematopoetic elements were found on the edge of sponges, no bone or cartilage tissue was detected (Figure 2H, S1). However, histology showed the tdTomato tagged Prx1-derived cells were present and survived (Figures 2I,J). Similarly, no bone or cartilage tissue formed with the periosteal implant of the donor mice transplanted to the intramuscular pouch of the wild-type B6 mice (Figure 2K, S1). Again, the tdTomato tagged Prx1-derived cells were present within the sponge and survived surgery (Figures 2L,M). Varying the amount of BMP2 did not induce ectopic bone formation for the transplant study with the different tissue sites (Figure S2c,d).

**FIGURE 3:**
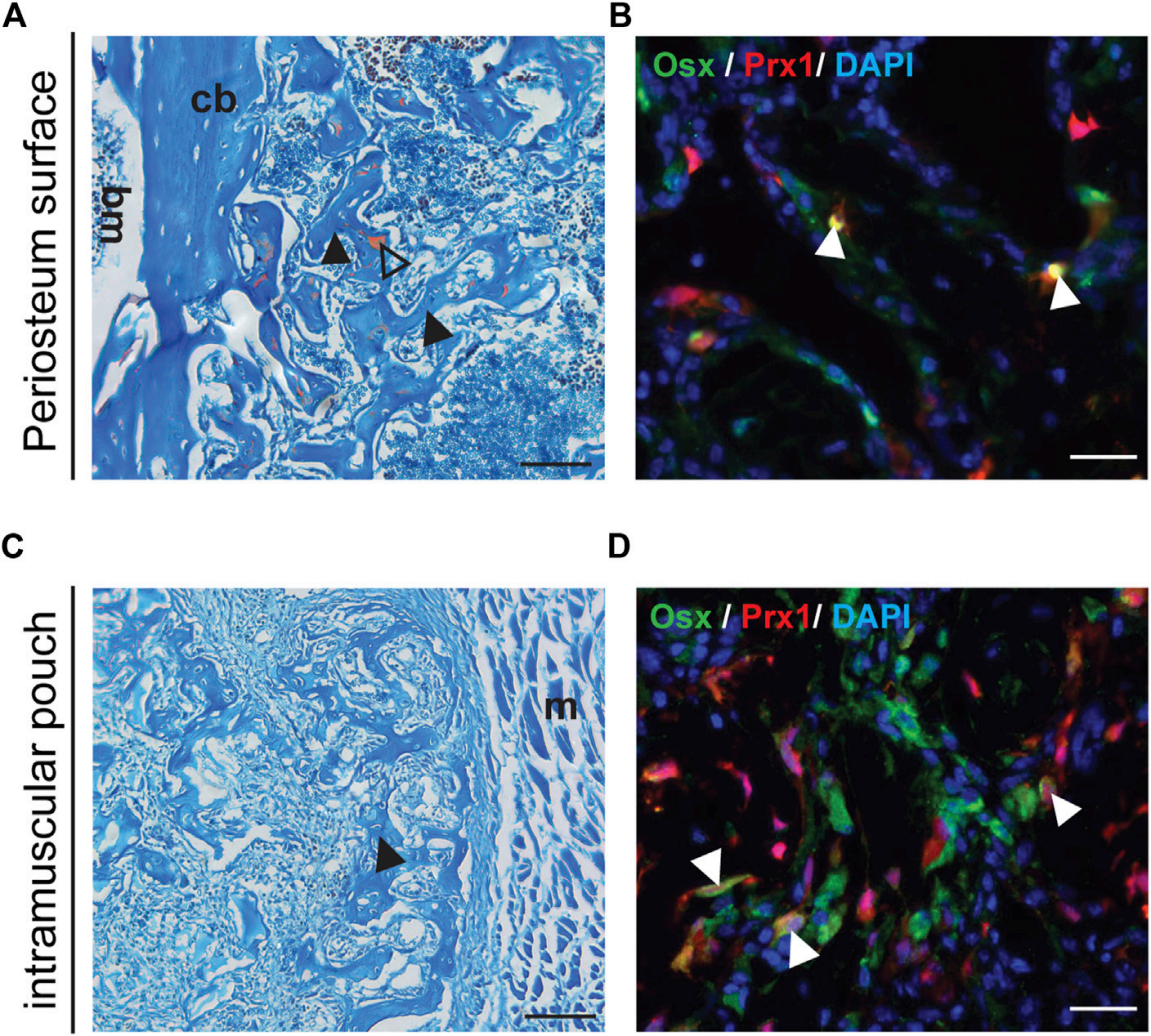
Transplanted Muscle-derived cells were able to form bone on periosteal surface and in muscle pouch with 5ug BMP2. Representative Safranin O and IF staining of the 5 µg BMP2 treated sponge with surrounding tissue at Day16 post-transplantation. Safranin O staining **(A, C)**, the black arrowheads indicate bone formation within the sponge, triangle shows cartilage formation. IF staining **(B, D)**, the white arrowheads indicate co-labeling of Prx1 derived cells (red) with osterix (green), nuclei were labeled with DAPI (blue). **(A, B)**. Results of muscle cells from Prx1 reporter mice transplanted to the periosteum surface of wild-type hosts. **(C, D)**. Results of muscle cells from Prx1 reporter mice transplanted to the muscle pouch of wild-type hosts. Scale bar = 100 μm for Safranin O, 25 µm for IF staining; bm, bone marrow; cb, cortical bone; m, muscle. Images are representative of 3 independent experiments.

Previous results suggested that increased amounts of BMP2 were needed to induce ectopic bone formation within the muscle using a gelatin sponge (Bragdon et al., 2017). Therefore 5 µg of BMP2 was used with the gelatin sponge implanted into the muscular pouch followed by transplant to either a muscular pouch or periosteum surface of the wild type B6 host mice. Following transplantation, bone tissue was detected at both the periosteal surface and within an intramuscular pouch (Figures 3A,C). The tdTomato tagged Prx1-derived cells differentiated into osteoblasts and contributed to ectopic bone formation (Figures 3B,D). These results suggest that BMP2 is an extrinsic factor that is crucial to the Prx1 cell population, intrinsic regulation on BMP2 sensitivity also plays a role in cell fate determination.

### Molecular profiling of the Prx1 cells from different skeletal tissues

**FIGURE 4:**
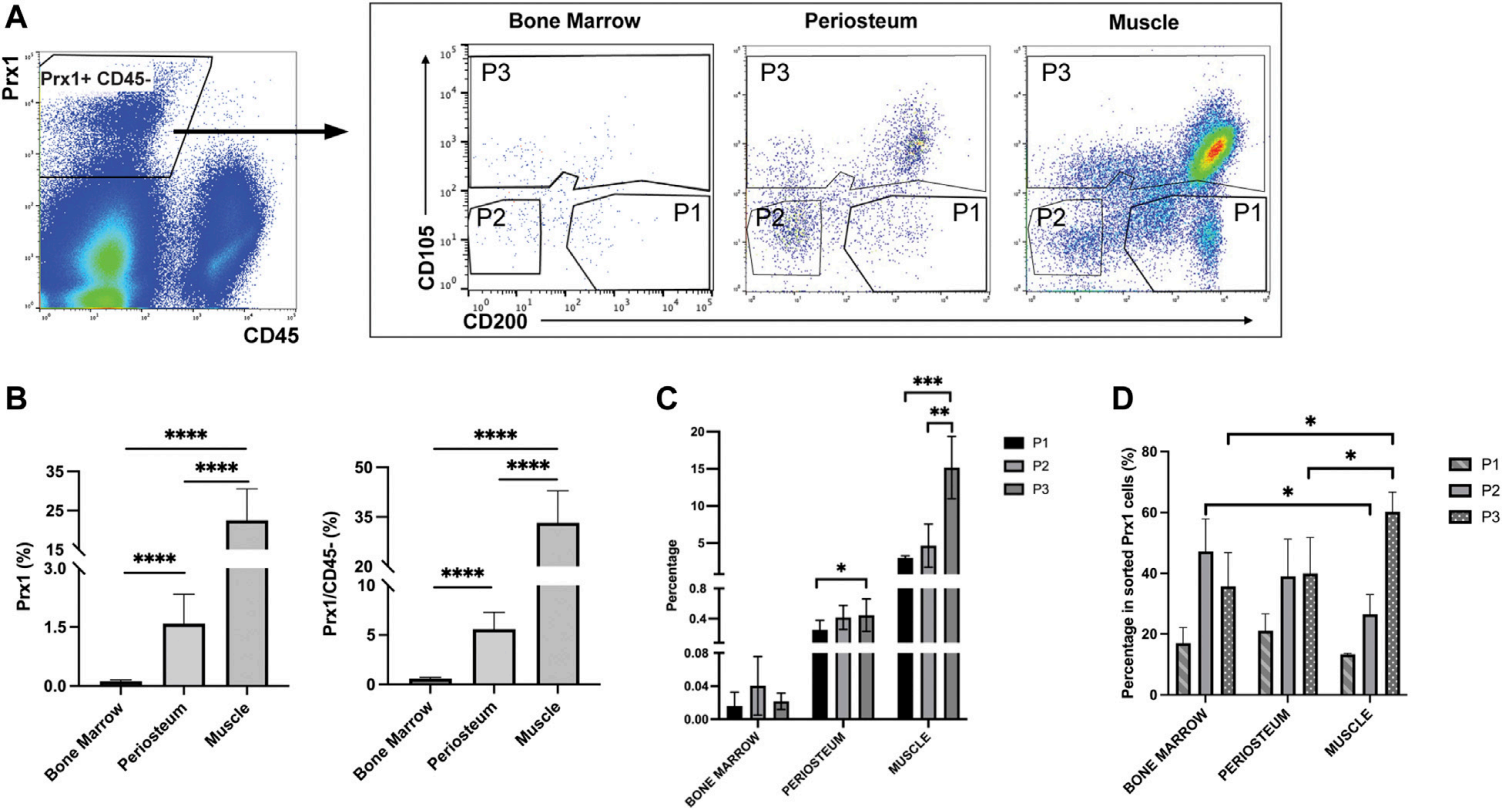
Flow cytometry analyses of bone marrow, periosteum and muscle cells isolated from Prx1CreER-GFP/Ai14/Rag1mice. **(A)**. Prx1-derived cells were harvested based on tdTomato expression. After excluding CD45^+^ cells, 3 subpopulations were subsequently isolated based on CD105 and CD200 expression. **(B)**. Quantification of Prx1+ cells harvested from bone marrow, periosteum and muscle. The percentages were calculated based on live cells or CD45-cells. **(C)**. Percentage of P1, P2, P3 cells of all live cells harvested from bone marrow, periosteum and muscle. **(D)**. Percentage of P1, P2, P3 cells of Prx1+ population harvested from bone marrow, periosteum and muscle. One-way ANOVA was performed followed by Tukey test to assess statistical significance. *p < 0.05, **p < 0.01, ***p < 0.001, ****p < 0.0001. *n* = 6. Data represent mean ± SD.

Prior to investigating the intrinsic differences between the Prx1 expressing cell populations of the periosteum and muscle, fluorescence-activated cell sorting (FACS) was used to determine the heterogeneity of the cell population. In addition to periosteum and muscle, Prx1-expressing cells were also isolated from the bone marrow, the cell population most prevalently used to investigate multi-potent cells (Figure 4A). Results showed the percentage of tdTomato tagged Prx1 derived cells were significantly higher from the muscle tissue (22.49 ± 8.03%) in comparison to periosteum (1.58 ± 0.75%) and bone marrow (0.1155 ± 0.04%). Considering the abundant number of hematopoitic cells in the bone marrow, the Prx1 percentage was also calculated based on CD45-population. Within the non-hematopoietic population isolated from the muscle, periosteum and bone marrow, the percentage of Prx1 cells were 33.22 ± 9.70%, 5.58 ± 1.70%, and 0.58 ± 0.13% (Figure 4B). Overall, Prx1-expressing cells have the highest fraction in skeletal muscle, which aligns with the observations we obtained from our previous histological analysis (Bragdon et al., 2022).

Recently, several cell surface proteins have been identified and utilized to isolate and enrich unique subsets of SSC/progenitor cells from different tissue niches and have included CD200, CD105, along with ITGAV, Cathepsin K (Chan et al., 2009; Chan et al., 2015; Gulati et al., 2018). These studies indicate that CD200+ CD105-cells represent the SSC population, while CD105+ and CD105-CD200-cells represent more committed populations. To further explore the heterogeneity of Prx1-derived cell populations, CD105 & CD200 were used as additional markers to distinguish Prx1 subsets of cells. Fractionated Prx1-tdTomato cells were enriched through the negative selection of hematopoietic cells (CD45^−^), then three subsets of skeletal stem and progenitor populations were isolated and used for analysis, P1 (CD105- CD200+), P2 (CD105- CD200-) and P3 (CD105+ CD200 variable) (Figure 4A). Percentage of Prx1 subsets followed the same trends as Prx1 population in different tissues, with highest percentages in muscle and lowest in bone marrow (Figure 4C). Overall, P1 showed the lowest proportion of Prx1-derived cells among all tissues (Figure 4D). Both Prx1 and the P1 subpopulation had the largest distribution in muscle.

**FIGURE 5:**
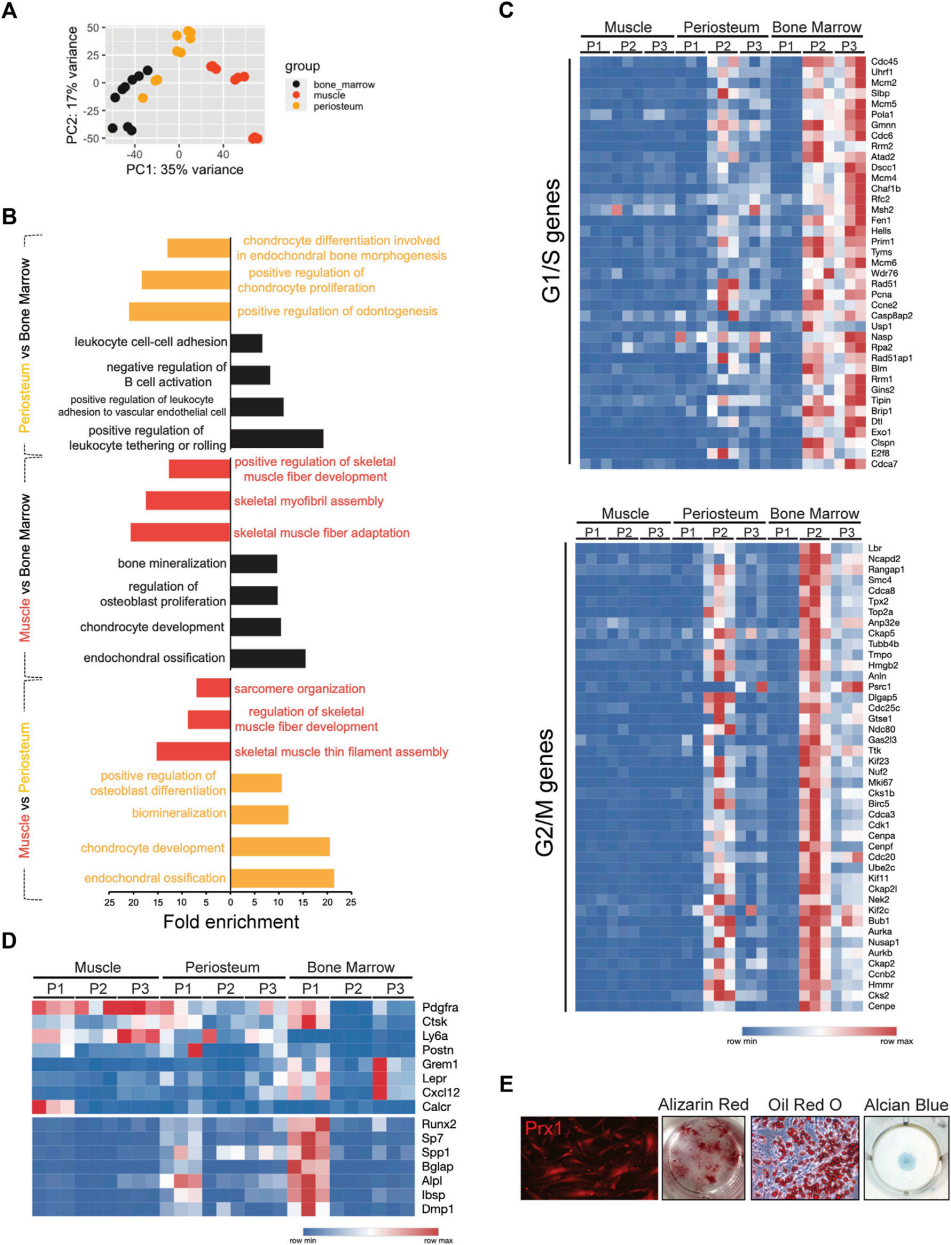
Bulk RNA-seq analysis of Prx1-derived cells from bone marrow, periosteum, and muscle. **(A)**. Principal component analysis of RNA-seq following FACS of Prx1-derived three subpopulations from bone marrow, periosteum and muscle tissues. *n* = 3 mice per group. **(B)**. GO analyses of Prx1 cells from three tissues: periosteum vs. bone marrow, muscle vs. bone marrow, muscle vs. periosteum, respectively. **(C)**. Heatmap of expression of the cell cycle-related genes in Prx1 subpopulations from bone marrow, periosteum and muscle. **(D)**. Heat map of gene expression in Prx1 subpopulations from bone marrow, periosteum and muscle. **(E)**. Images of cultured muscle-resident Prx1-derived P1 cells. Left to right: images of cells at Day21 post-harvest; Alizarin red, oil red O and alcian blue staining of cells. N = 5

Next, bulk-RNA sequencing analysis was utilized to define the distinct transcriptomic properties of the Prx1-derived cells. Transcriptional analysis showed the Prx1-derived cells harvested from the three tissues were broadly different (Figure 5A). Additionally, gene ontology (GO) analysis for biological processes of three tissues suggested that terms indicating ossification, mineralization and bone formation were more commonly associated with bone marrow and periosteum when compared with muscle. Further comparison between bone marrow and periosteum revealed bone marrow-derived Prx1 cells were enriched in GO categories related to “immune response”, indicating a strong immunological modulation ability (Castillo et al., 2007; Jiang and Xu, 2020) (Figure 5B). Collectively, these findings reveal that skeletal stem/progenitor cells from different tissues have distinct molecular profiles and periosteum are more active in bone formation at homeostatic conditions.

To achieve a better understanding of the similarities and differences between the subsets of Prx1-derived cells at the transcriptomic level, comparison between subpopulations was performed. Besides tissue dissimilarities, analysis of Prx1 subpopulations demonstrated that Prx1-derived cells are heterogeneous, in which three subpopulations clustered separately based on principal component analysis (PCA) and expressed distinguished sets of genes (Figure S4), indicatingthe identification of truly distinct subpopulations of the Prx1 cell populations isolated from three separate tissues. We next performed GO analysis that compared P1 *versus* progenitor populations P2 and P3 across all tissues, results showed P1 expressed “ossification” and “osteoblast differentiation” gene sets, suggesting these cells play an important role in bone formation. In contrast, P2/P3 were enriched in GO categories, such as “cell cycle”, “DNA-replication-dependent nucleosome assembly” and “cell division” (Figure S5a). To gain further understanding of cell-cycling features, transcriptomes were compared to available datasets assembled from the signatures of cell-cycle G1/S and G2/M phases (Tirosh et al., 2016). We found that P1 in all three tissues were quiescent with low expression in G1/S and G2/M genes. By contrast, P2/P3 from bone marrow and periosteum were relatively active (Figure 5C). Remarkably, all Prx1 subpopulations from muscle did not show cell proliferation, suggesting an exit from the cell cycle that is seen with quiescence.

Common cell markers of mesenchymal stem/progenitors and osteogenesis were used as a comparison. Markers previously known to define mesenchymal marrow stromal cells such as Gremlin1, Cxcl12, Leptin R, periosteal progenitor makers, Cathepsin K, Postn, and markers universally associated with all sources of MSCs, PDGFRα, Ly6a were included. Muscle Prx1 subpopulations did not express osteogenic genes equal to the periosteal or bone marrow derived cells but exhibited high expression of mesenchymal stem/progenitor cell markers, suggesting they remain at a primitive state without differentiating. By contrast, the P1 cells of the periosteum and bone marrow showed enrichment of several known osteogenic genes, such as runx2, sp7, bglap, alpl, *etc.* The results demonstrated a large overlap with common mesenchymal stem/progenitor markers (Figure 5D). When compared with progenitor populations of muscle, P1 expressed cartilage development, ossification and tissue development GO terms (Figure S5b). *In vitro* culture confirmed multipotency of muscle-derived P1 cells with differentiation into osteoblasts, adipocytes and chondrocytes (Figure 5E). Thus, our results suggest muscle-derived SSCs possess unique properties when compared with other skeletal tissues.

### Muscle-derived Prx1 cells are quiescent but become activated with BMP2 and injury

**FIGURE 6:**
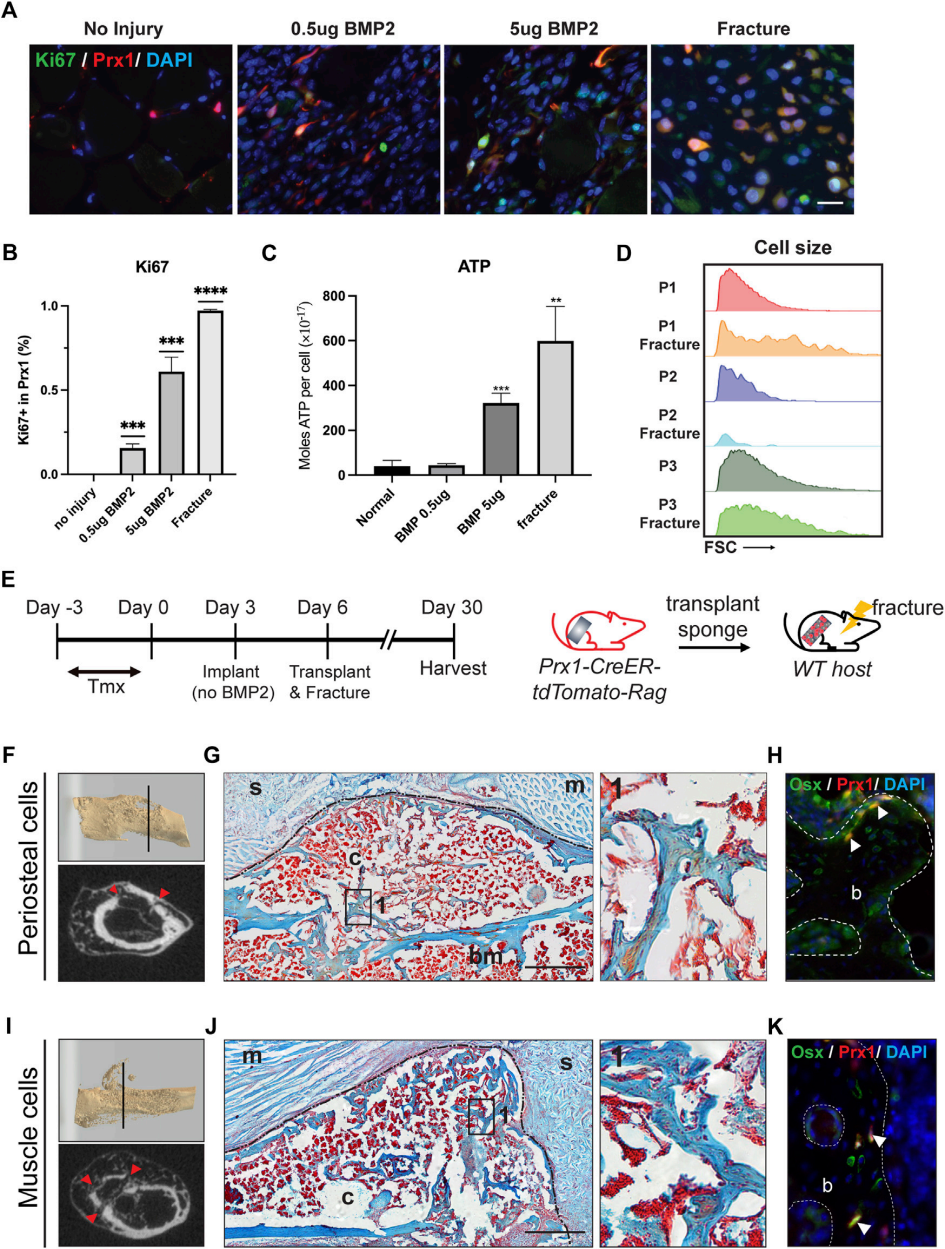
BMP2 and fracture activate muscle-derived Prx1 cells. **(A)** Co-labeling of Prx1 derived cells (red) with Ki67(green) in normal, BMP2 0.5µg, BMP2 5 µg and fracture conditions. Nuclei were labeled with DAPI (Blue). Scale bar = 25  μm **(B)**. Quantification of Prx1-derived cells that are Ki67+ in normal, BMP2 0.5µg, BMP2 5 µg and fracture conditions. **(C)**. P1 isolated from fracture injured and BMP2 5 µg treated muscle display elevated ATP level than normal and BMP2 0.5 µg groups. One-way ANOVA was performed followed by Tukey test to assess statistical significance. **p < 0.01, ***p < 0.001, ****p < 0.0001. n = 3. Data represent mean ± SD. **(D).** Compared with two other subsets, P1 subpopulations displayed a significant increase in cell size following fracture, as measured by the FSC parameter by FACS. **(E)**. Schematic representation of sponge transplantation with femur fracture. **(F–H)**. Results of periosteum cells from Prx1 reporter mice transplanted to the fracture femur of wild-type hosts. **(I–K)**. Results of muscle cells from Prx1 reporter mice transplanted to the fracture femur of wild-type hosts. **(F, I)**. Representative micro-CT of fracture calluses and surrounding tissue at Day24 post-transplantation. Top: 3D reconstruction of fractured femur. Bottom: micro-CT slice images, red arrowhead indicates fracture sites. **(G, J)**. Longitudinal section of the sponge and femur, stained with Safranin O. Black dotted lines represent boundaries of fracture callus. Right: Enlarged view of boxed region 1 showing bone formation. **(H, K)**. Adjacent section immunostained with osterix (green). Nuclei were labeled with DAPI (blue). The white arrowheads indicate co-labeling of Prx1 derived cells (red) with osterix. Scale bar = 500 μm for Safranin O, 50 µm for IF staining. B = bone; bm = bone marrow; c = callus; s = sponge; m = muscle. *n* = 3.

Since the muscle-derived Prx1 cells maintained an overall quiescent state but still possessed osteogenic capacity, we investigated the level of activation of the muscle-derived Prx1 cells and if activation can lead to bone formation. Upon activation, quiescent adult stem cells transit into an activated state with increased proliferation, cell size, and ATP levels (Freter et al., 2010; Rodgers et al., 2014). Ki67 is used to label proliferating cells as it is a cell cycle protein specifically expressed in G1-S-G2-M phases of the cell cycle but is absent in the G0 phase (Gerdes et al., 1984; Scholzen and Gerdes, 2000; Hutchins et al., 2010). Immunofluorescent staining for ki67 confirmed that proliferating tdTomato tagged Prx1-derived cells were absent within the muscle. The implantation of the gelatin sponge with 0.5 µg BMP2, 5 µg BMP2, and fracture significantly increased the number of Ki67 + Prx1-derived cells with a dose dependent response (Figures 6A,B). Keeping with the *in vivo* results, we found that injury and 5 µg BMP2 increased levels of cellular ATP of the P1 subpopulation, which align with the previous results that only the 5 µg BMP2 could induce ectopic bone formation within the muscle. While stimulations with low amounts of BMP2 (0.5 ug) did not fully induce stem cell activation (Figure 6C). Additionally, the P1 subpopulations displayed a significant increase in cell size following injury relative to the quiescent state at homeostasis (Figure 6D). Collectively, these data indicate that quiescent muscle cells require greater stimulations to be active and prepare the cells for regeneration.

Following injury, the quiescent adult stem cells respond to external stimuli and become active. The periosteal-derived Prx1 cells within adult bones have been shown to participate in bone repair (Logan et al., 2002; Kawanami et al., 2009), however, few studies investigated how muscle-derived stem cell/progenitors participate in this process. Julien et al. showed muscle-derived Prx1 cells mediate the initial fibrotic response to bone injury and also participated in cartilage and bone formation (Julien et al., 2021). In our experiment, the femoral fracture model applies a blunt trauma onto the femur creating a closed, simple transverse fracture while inducing injury to both the muscle and femur. To investigatethe activity of the Prx1 cells in response to injury, especially how injury influence muscle Prx1 cells, gelatin sponges were transplanted from either the periosteum or muscle to the fracture site of the host animal. After 24 days the callus was formed and bridging occurred at the fracture site, as shown by micro-CT and Safranin O staining (Figures 6F,G,I,J). Both the periosteal and muscle Prx1-derived cells were detected within the callus and labeled with the osteogenic marker, Osterix. The transplanted cells survived surgery and injury, migrated to the fracture callus, and contributed to callus formation (Figures 6H,K). Collectively, these data indicate that muscle Prx1 cells are quiescent stem cells and require stimulation such as high levels of BMP2 or injury to become active and osteogenic.

## Discussion

In this study, the heterogeneity of a mesenchymal cell population marked by Prx1 expression was established across and within different tissues. Multiple features were examined, including transcriptomic differences in these cells and the extrinsic features such as the role of the local tissue environment and functional ability to differentiate in response to BMP2 signaling and fracture. Together, our analyses of homeostatic skeletal tissues, BMP2-induced ectopic bone formation, and injury conditions highlighted the distinct functional and molecular properties of muscle-resident skeletal stem cells, which are maintained in quiescent state but can be activated upon BMP2 and fracture stimulations.

The transplant study demonstrated the muscle environment is not permissive for bone formation under homeostasis, while the periosteum creates a supportive niche for SSCs to participate in bone remodeling and maintenance. Although the muscle derived Prx1 cells can differentiate toward the osteogenic lineage *in vitro*, these cells are not osteogenic when transplanted to a permissive bone-forming environment of the periosteum and only form bone *in vivo* when higher BMP2 concentrations are present relative to periosteal cells. These observations suggest that muscle cells possess an intrinsic regulatory mechanism allowing for an osteogenic capacity but requiring specific stimulation(s) to commit to the osteogenic fate. Of note, the true functional differences that cells exhibit *in vivo* provide more information on better understanding how cell behave in more physiological conditions and are not always fully seen under an artificial *in vitro* setting. Such *in vivo* and *in vitro* discrepancy is observed in many similar studies. For example, Greenblatts et al. (Debnath et al., 2018) transplanted periosteum stem cells (Cathepsin K+) and non-Cathespin K mesenchymal stem cells under the kidney capsule of wild-type recipient mice, showed periosteum stem cells underwent intramembranous bone formation while non-CathespinK MSCs were able to form cartilage, however both populations of cells showed tri-lineage differentiation capacities when culture *in vitro*. The finding of bone-forming cells in skeletal muscle is consistent with Chan et al. (Chan et al., 2015), who demonstrated unknown progenitor cells within the extra skeletal tissues can differentiate with BMPs. In addition, these muscle-resident mesenchymal cells have been shown to mineralize in bone repair, based on our findings and others (Julien et al., 2021). Previous studies (Oishi et al., 2013; Eisner et al., 2020; Leinroth et al., 2022) identified PDGFRα+ cells as the pathologic cell type associated with HO. The muscle Prx1-derived cells showed a high expression of PDGFRα indicating the muscle Prx1-derived cells may be responsible for HO formation through a mechanism of activation and reviving their skeletogenic potential following pathological stimulus. Thus, our findings about muscle-resident mesenchymal cell population indicate their broad implications in understanding and treating skeletomuscular diseases and injury.

RNAseq analyses reveal that Prx1-derived population itself is heterogeneous and identified in this study are three distinct subpopulations. P2 and P3 subpopulations are enriched with cell-cycle related GO terms, indicating active cell proliferation and division, which is consistent with previous studies showing CD105-CD200-and CD105+CD200^variable^ labels skeletal progenitor cells that are more fate-limited and active in differentiation (Chan et al., 2015). Unsurprisingly, P1 subpopulation express markers previously shown to define mesenchymal stem cell, such as Ly6a, Ctsk. However, P1 also showed overexpression of osteogenic-related genes. Recent studies (Matthews et al., 2021) found CD200 not only can identify skeletal stem cells but also labels mature osteogenic cells, raising the possibility that our P1 population contains osteoblasts/osteocytes. This overexpression is observed in all three tissues where bone marrow shows the most significant difference while muscle to a much less extent. It implies that muscle-derived skeletal stem/progenitor cells do not demonstrate osteogenic capacity under homeostasis unless they receive environmental signals, where bone marrow and periosteum counterparts are constantly turned over at a higher rate and involved in homeostatic maintenance of the bone tissue as previously suggested (Park et al., 2012; Bragdon et al., 2022).

Quiescence is a unique property in which many adult stem cells exist in a non-proliferative state. Stem cells are elaborately maintained in the reversible G0 state but poised for activation, they can transiently re-enter the cell cycle to proliferate and differentiate (Urbán and Cheung, 2021). Although this aspect of stem cell biology has been extensively studied in cell types like muscle satellite cells or neural stem cells (Matsubara et al., 2021; Brunet et al., 2022; Sousa-Victor et al., 2022), the regulation of the quiescent state in mesenchymal stem cells remains largely unexplored. Here we show there is a diversity of quiescence among Prx1-derived cells in different tissues. P2/P3 from bone marrow and periosteum display high propensity to cycle, which enable a rapid response to bone injury and tissue homeostasis. In contrast, skeletal muscle maintenance and repair is believed to be mainly regulated by satellite cells, which can proliferate and differentiate into myoblasts. Though skeletal stem cells are abundant in healthy muscle, our work indicates these quiescent Prx1-derived cells serve as a reserve pool of stem cells which are distinct from satellite cells, and their dormancy is only reversed by strong environmental or injury-induced signals. Recent literature (Joe et al., 2010; Julien et al., 2021) suggests they can also rapidly differentiate into fibroblasts in response to acute muscle damage, which infers their role in regeneration and how environment controls their fate decisions. However, the molecular mechanisms that regulates functionally distinct states require further investigation. Several studies highlighted the role of G-protein-coupled receptors (GPCRs) -mediated signaling in maintaining quiescence of adult stem cells (Chagin et al., 2014; Codega et al., 2014). Notably, calcitonin receptor (Calcr), a GPCR receptor, is reported to keep muscle satellite cells in their quiescent state (Yamaguchi et al., 2015). Among three muscle Prx1-derived subpopulations, only stem cell population P1 showed a high expression of Calcr than the other progenitor subsets, strongly suggesting that muscle SSCs are also regulated in a quiescent state through Calcr (Figure 5D). Besides, BMP2 is an essential mediator of the endogenous bone repair response, which function cannot be compensated by other stimuli (Tsuji et al., 2006). Here, we show that both exogenous BMP2 and fracture-induced endogenous BMP2 can promote the activation of muscle SSCs, indicating BMP2 signaling pathway may serve as a negative regulator of SSC quiescence.

Collectively, the data we present here demonstrate the Prx1 cell population is highly diverse between skeletal tissues and within the tissues they reside. Skeletal muscle stem cells are abundant in the tissue, can quickly enter cell cycle in response to external stimuli. Since muscle skeletal stem cells yield the cellular pool required for bone regeneration, it provides the opportunity that manipulating their activity may provide new opportunities to treat orthopedic diseases. A better understanding of skeletal stem cell quiescence and the intrinsic mechanisms by which cells respond to environmental signals will undoubtedly aid the design of new therapeutic approaches based on enhancing stem cell functionality.

## Data Availability

The datasets presented in this study can be found in online repositories. The names of the repository/repositories and accession number(s) can be found below: https://www.ncbi.nlm.nih.gov/geo/, GSE218550.
